# Surface area of particle administered versus mass in determining the pulmonary toxicity of ultrafine and fine carbon black: comparison to ultrafine titanium dioxide

**DOI:** 10.1186/1743-8977-6-15

**Published:** 2009-05-04

**Authors:** Tina M Sager, Vincent Castranova

**Affiliations:** 1 Health Effects Laboratory Division, National Institute for Occupational Safety and Health, Morgantown, West Virginia, USA; 2 Department of Environmental Health, Harvard School of Public Health, Boston, Massachusetts, USA

## Abstract

**Background:**

Nanoparticles are characterized by having a high surface area per mass. Particulate surface area has been reported to play an important role in determining the biological activity of nanoparticles. However, recent reports have questioned this relationship. This study was conducted to determine whether mass of particles or surface area of particles is the more appropriate dose metric for pulmonary toxicity studies. In this study, rats were exposed by intratracheal instillation to various doses of ultrafine and fine carbon black. At 1, 7, or 42 days post-exposure, inflammatory and cytotoxic potential of each particle type was compared on both a mass dosage (mg/rat) as well as an equal surface area dosage (cm^2 ^of particles per cm^2 ^of alveolar epithelium). In an additional study, the pulmonary responses to instillation of ultrafine carbon black were compared to equivalent particle surface area doses of ultrafine titanium dioxide.

**Results:**

Ultrafine carbon black particles caused a dose dependent but transient inflammatory and cytotoxic response. On a mass basis, these responses were significantly (65 fold) greater than those for fine sized carbon black. However, when doses were equalized based on surface area of particles given, the ultrafine carbon black particles were only slightly (non-significantly) more inflammogenic and cytotoxic compared to the fine sized carbon black. At one day post-exposure, inflammatory potencies of the ultrafine carbon black and ultrafine titanium dioxide particles were similar. However, while the pulmonary reaction to ultrafine carbon black resolved with time, the inflammatory effects of ultrafine titanium dioxide were more persistent over a 42 day post-exposure period.

**Conclusion:**

These results indicate that for low toxicity low solubility materials, surface area of particles administered rather than mass burden of particles may be a more appropriate dose metric for pulmonary toxicity studies. In addition, ultrafine titanium dioxide appears to be more bioactive than ultrafine carbon black on an equivalent surface area of particles delivered basis.

## Background

Nanotechnology is considered to be one of the world's most promising new technologies, able to impact all phases of life, just as the industrial revolution did in the past two centuries. Utilizing the quantum properties of atoms and molecules, nanotechnology proposes the construction of novel molecular devices possessing extraordinary properties. However, both epidemiological and toxicological studies have contributed to a body of evidence suggesting that nano or ultrafine particles may induce or exaggerate a number of adverse biological effects. It has been suggested that nanoparticles may interfere with a number of molecular processes that should be considered before such particles are brought into wide commercial use [1].

Recent reports indicate that there can be considerable potential for exposure to nanoparticles in the workplace, especially during transfer, weighing, blending, and cleaning processes [2,3]. There is also interest and debate as to whether low solubility, ultrafine particles should be regulated differently from fine size particles of the same composition [4].

When evaluating exposure-dose-effect relationships of inhaled particles, the definition and determination of dose is crucial. Conventionally and conveniently, doses usually are expressed in terms of particle mass. However, when the pulmonary inflammatory potential of ultrafine TiO_2 _was compared to fine size TiO_2_, particle surface area was found to be a better predictor of bioactivity than particle mass burden delivered [5]. Studies, such as this with nanoparticles, have been used to support the hypotheses that particle surface area is important in determining pathology [6] and inflammation [7]. However, recent reports by Warheit et al. [8,9] have questioned this hypothesis.

In general, for a fixed mass of particles, surface area increases as particle size becomes smaller. Thus, a dose-dependence on particle surface area may explain the greater toxicity of nanoparticles compared with an equal mass of fine particles of the same material [10,11]. The finding that particle surface area rather than mass appears to be a more appropriate metric of dose for predicting pulmonary inflammation may imply a need to reconsider exposure assessment practices for workplaces producing or using nanoparticles. Currently, occupational exposure limits for airborne dusts are defined in terms of mass per m^3 ^of air [11]. In support for the importance of exposure standards, Serita et al. [12] conducted an experiment that exposed rats to metallic ultrafine nickel at the Japanese regulated occupational exposure level (OEL). This OEL was based on data for fine nickel particles. However, the exposure to OEL concentrations in the form of ultrafine nickel caused severe lung injury after a single exposure [12]. This finding supports the concept that surface area is the dose measure that predicts pulmonary response, rather than mass, and this has far reaching potential consequences for occupational standards that are based on mass [13].

Therefore, if in fact the inflammatory potential of a particle is driven by surface area rather than mass, then a given airborne mass concentration of a material in the form of fine particles could be much less inflammogenic than the same airborne mass concentration of same material in the form of ultrafine particles. Therefore, determining if high surface area of nanoparticles is a driver for inflammatory potential is of great importance for development of protective occupational health measures.

The present study aims to address the issue of whether, for low toxicity low solubility materials, mass or surface area of particles administered is a more appropriate dose metric when assessing pulmonary toxicity of nanoparticles. To address this question, in vivo intratracheal exposures of rats to ultrafine carbon black (UFCB) and fine carbon black (FCB) were conducted. Animals were exposed to carbon black particles based on mass, and dose was normalized to surface area of particles administered. Resulting pulmonary damage and inflammation were compared on a mass dose and surface area of carbon black particles administered to determine which dose metric was more appropriate in evaluating nanoparticle toxicity. In additional experiments, the pulmonary activity of ultrafine carbon black (UFCB) was compared to ultrafine titanium dioxide (UFTiO_2_) on an equivalent surface area of particles delivered basis.

## Results

UFCB and FCB suspended in acellular bronchoalveolar lavage fluid (BALF) were administered to Fischer 344 rats via intratracheal instillation to assess pulmonary toxicity. The dose of particles administered was given on a mass basis (mg/rat) and was also normalized to surface area of particles administered per alveolar epithelial surface area (cm^2^/cm^2^) using a value for alveolar epithelial surface area in the rat reported by Stone et al. [14]. The surface areas of the respective particles (269.0 m^2^/g for UFCB and 8.1 m^2^/g for FCB, respectively) were determined by the BET gas absorption technique [15]. This comparison of mass and surface area doses was conducted to assess whether surface area of particles administered is the more appropriate dose metric that should be considered when assessing nanoparticle pulmonary toxicity parameters. Pulmonary toxicity parameters measured included PMN number, LDH activity, albumin levels, inflammatory mediators (TNF-α, MIP-2, and IL-1β), as well as zymosan-stimulated chemiluminescence and NO dependent chemiluminescence.

### Comparison of pulmonary toxicity of UFCB and FCB

**Figure 1:**
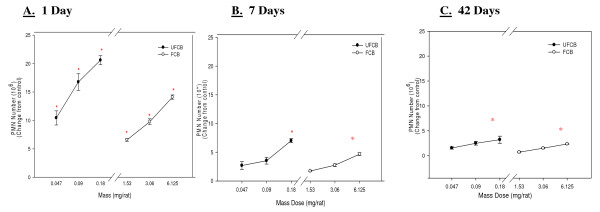
**Comparison of inflammation elicited in animals receiving various mass doses of UFCB and FCB suspended in BALF**. A comparison of inflammation elicited in animals receiving various mass doses of UFCB and FCB suspended in BALF at 1 day (Panel A), 7 days (Panel B), and 42 days (Panel C) post-exposure. Rats were exposed to various mass doses of UFCB and FCB by intratracheal instillation. Animals were euthanized at 1 day, 7 days, and 42 days post-exposure, and bronchoalveolar lavage was performed. Inflammation was assessed by BAL PMN counts. Values are increased PMN number above the BALF control and are given as means ± SEM of 8 rats. Control PMN values were 1.45 ± 0.22 × 10^6^, 1.09 ± 0.14 × 10^6^, and 1.01 ± 0.14 × 10^6 ^cells/rat for 1, 7 and 42 days respectively. Linear regression analysis with a 95% confidence interval reveals that on a mass dose basis UFCB causes significantly more inflammation than FCB at all post-exposure time points. On a mass dose basis, UFCB is shown to be approximately 65 times more potent than the FCB at all post-exposure time points. * indicates a significant increase from control (p < 0.05; ANOVA).

UFCB and FCB both caused a dose dependent increase in the number of PMN obtained by BAL over the 42 day post-exposure time period (Figure 1). The inflammatory response for both UFCB and FCB was transient, decreasing strikingly with time. However, the high dose of UFCB and FCB still caused a significant increase in PMN number over control at 1, 7 and 42 days post-exposure (Table 1). Table 1 shows comparison on pulmonary toxicity parameters measured in control animals and for animals receiving the high doses (0.18 and 6.125 mg/rat) of UFCB and FCB, respectively, at all post-exposure time points. At 1 day, 7 days and 42 days post-exposure, on a mass dose basis, UFCB was significantly more inflammogenic than FCB (Figure 1). When comparing the inflammogenic response of UFCB exposure to FCB exposure, on a mass dose basis (for example the PMN response to 3.06 mg/rat FCB to 0.047 mg/rat UFCB from Figure 1), UFCB was shown to be 65 times more potent than FCB at all post-exposure times (Table 2). Table 2 shows The data were analyzed to show the potency difference between UFCB and FCB on a mass basis as well as the fold increase in pulmonary toxicity response on a surface area basis, ie., surface area of particles administered per surface area of alveolar epithelium where the value for the alveolar epithelial surface area for the rat was taken from Stone et al. [14]. All post-exposure time points were analyzed and are reported in the table. On a mass basis, the UFCB has much greater potency than FCB, but when dose is normalized to particle surface area administered the fold increase in response between the UFCB and FCB is greatly reduced.

**Table 1:** Effect of Exposure to UFCB vs. FCB (High Dose) on pulmonary responses.

**Parameter**	**Group**	**1 day**	**7 days**	**42 days**
PMN (10^6^)	Control	1.5 ± 0.2	1.1 ± 0.1	1.0 ± 0.1
	UFCB high	22.1 ± 0.8*	8.1 ± 0.3*	4.2 ± 0.7*
	FCB high	15.5 ± 0.4*	5.7 ± 0.2*	3.3 ± 0.2*
				
LDH (U/l)	Control	49.3 ± 1.4	43.8 ± 0.8	45.1 ± 1.7
	UFCB high	179.1 ± 6*	116.3 ± 4*	84.6 ± 2*
	FCB high	135.3 ± 6*	88.6 ± 2*	71.9 ± 1*
				
Albumin (mg/ml)	Control	0.21 ± 0.1	0.12 ± 0.01	0.18 ± 0.02
	UFCB high	0.48 ± 0.02*	0.31 ± 0.025*	0.32 ± 0.01*
	FCB high	0.40 ± 0.01*	0.25 ± 0.01*	0.26 ± 0.01*
				
TNF-α (pg/ml)	Control	24.8 ± 0.5	28.2 ± 0.6	32.2 ± 0.6
	UFCB high	45.3 ± 2*	50.4 ± 1*	60.1 ± 1*
	FCB high	30.9 ± 0.8	34.3 ± 1	38.6 ± 0.9
				
MIP-2 (pg/ml)	Control	437.7 ± 14	497.2 ± 22	476.5 ± 22
	UFCB high	544.1 ± 34*	727.8 ± 29*	615.2 ± 21*
	FCB high	384.9 ± 22	485.8 ± 22	532.9 ± 22
				
IL-1β (pg/ml)	Control	58.1 ± 4	69.7 ± 5	70.5 ± 7
	UFCB high	184.9 ± 10*	213.6 ± 8*	262.8 ± 9*
	FCB high	78.6 ± 6	83.7 ± 9	115.7 ± 12
				
Zym. Stim. Chemi.	Control	3.14 ± .08	3.06 ± .09	3.22 ± .30
	UFCB high	7.28 ± .13*	5.35 ± .12*	3.85 ± .55
	FCB high	4.48 ± .10*	4.06 ± .04*	2.96 ± 41
				
NO Dep. Chemi.	Control	0.68 ± .05	0.52 ± .06	0.49 ± .07
	UFCB high	4.2 ± .06*	3.6 ± .05*	2.4 ± .40*
	FCB high	4.0 ± .04*	1.7 ± .05*	1.3 ± .04

* denotes that the change from control is statistically significant (p < 0.05) using ANOVA.

**Table 2:** Potency difference between UFCB and FCB when analyzed on a mass vs. surface area.

**Parameter**	**Dose Metric**	**1 Day**	**7 Days**	**42 Days**
PMN (10^6^)				
	Mass	65	65	65
	Surface Area	2	1.5	1.6
LDH (U/l)				
	Mass	65	65	65
	Surface Area	1.5	2	1.8
Albumin				
	Mass	65	65	65
	Surface Area	1.5	2	2
TNF-α (pg/ml)				
	Mass	130	130	130
	Surface Area	1.4	1.5	1.5
MIP-2 (pg/ml)				
	Mass	130	130	130
	Surface Area	1.4	1.7	1.3
IL-1β (pg/ml)				
	Mass	130	130	130
	Surface Area	2.4	2.6	2.3
Zym. Stim. Chemi.				
	Mass	130	130	130
	Surface Area	1.5	1.75	1.2
NO Dep. Chemi.				
	Mass	68	130	130
	Surface Area	1.5	2.5	3

Comparison of UFCB and FCB potency differences.

**Figure 2:**
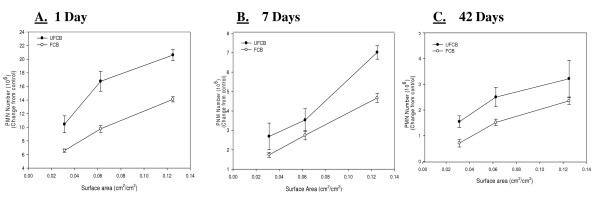
**Comparison of inflammation elicited in animals receiving UFCB and FCB normalized to surface area of particles administered per surface area of alveolar epithelium**. A comparison of inflammation elicited in animals receiving doses (0.0313, 0.0625 and 0.125 cm^2^/cm^2^) of UFCB and FCB normalized to surface area of particles administered per surface area of alveolar epithelium at 1 day (Panel A), 7 days (Panel B), and 42 days (Panel C) post-exposure. Particles were suspended in BALF. Alveolar epithelial surface area for the rat was taken from Stone et al. [14]. Rats were exposed to various doses of UFCB and FCB by intratracheal instillation. Animals were euthanized at 1 day, 7 days, and 42 days post-exposure and bronchoalveolar lavage was performed. Inflammation was assessed by BAL PMN counts. Values are increased PMN number above the BALF control and are given as means ± SEM of 8 rats. Control PMN values were 1.45 ± 0.22 × 10^6^, 1.09 ± 0.14 × 10^6^, and 1.01 ± 0.14 × 10^6 ^cells/rat for 1, 7 and 42 days respectively. Linear regression analysis with a 95% confidence interval reveals that when dose is normalized to surface area of particles administered, dose responses curves assessing inflammation caused by UFCB and FCB exposure are not significantly different. On a dose normalized to surface area UFCB elicits at most a 2 fold increase in inflammation when compared to FCB at all post-exposure time points.

However, when the dose of particles was normalized to surface area of particles administered the difference in inflammogenic responses, assessed by PMN number, of the two particle types became less. When comparing the dose response curves assessing inflammation produced by the UFCB and FCB exposures, normalized to surface area of particles, a linear regression curve analysis with a 95% confidence interval showed that there was no significant difference between the two dose-response curves at any post-exposure time (Figure 2). In fact, when dose was normalized to surface area of particles administered, the inflammogenic response elicited by UFCB was at most 2-fold greater than the FCB response for all post-exposure time points (Table 2).

In regards to the other pulmonary toxicity parameters assessed for UFCB and FCB exposure, the same trend was noted. LDH activity in BALF was measured to assess cellular cytotoxicity, and BALF albumin levels were analyzed to assess air/blood barrier injury. Exposure to either UFCB or FCB caused a dose dependent cytotoxicity which declined with time post-exposure (data similar to figure 1 not shown). However, at the high dose, LDH activity remained significantly elevated over control at all post-exposure time points (Table 1). Effects on albumin levels showed the same trend as LDH activity, with exposure to the high dose of both UFCB and FCB causing a significant increase in albumin levels over control at all post-exposure time points (Table 1). On a mass dose basis, a significantly greater mass dose of FCB (65 fold higher) was required at all post-exposure time points, to obtain the same responses as seen with UFCB exposure in regards to LDH activity and albumin levels (Table 2). However, when dose was normalized to surface area of particles administered, the UFCB exposure produced LDH activity and albumin levels that were at most only 2-fold greater than the FCB exposure at all post-exposure time points analyzed (Table 2). When dose-response curves assessing LDH activity and albumin levels based on surface area of particles administered between UFCB and FCB exposure were assessed using a linear regression curve analysis with a 95% confidence interval (data similar to figure 2 not shown), there was no significant difference between the two curves at any post-exposure time points.

IL-1β, TNF-α, and MIP-2 mediator levels in the BAL were also measured for UFCB and FCB exposure at all post-exposure time points. The highest dose of UFCB caused a significant increase in these mediator levels over control values at all post-exposure time points. However, FCB exposure did not increase mediator levels significantly over control at any post-exposure time point (Table 1). For all three mediators, a significantly greater mass dose of FCB (130 fold higher) was required to obtain the comparable mediator levels as elicited by UFCB exposure (Table 2). However, when dose was normalized to surface area of particles administered, TNF-α and MIP-2 levels produced by UFCB exposure were at most only 1.5 or 1.7 fold greater than FCB levels. IL-1β cytokine levels for UFCB exposure were at most only 2.6 fold greater than FCB IL-1β cytokine levels (Table 2). Dose response curves assessing TNF-α, IL-1β, and MIP-2 mediator levels based on surface area of particles administered between UFTiO_2 _and FTiO_2 _exposure were assessed using a linear regression curve analysis with a 95% confidence interval (data similar to figure 2 not shown). This analysis showed there was no significant difference for any of the mediators between the two curves at any post-exposure time point.

Alveolar macrophage zymosan-stimulated and NO dependent chemiluminescence were measured to assess reactive oxygen species production after UFCB and FCB exposures. UFCB and FCB both caused dose-dependent and transient increases in zymosan-stimulated chemiluminescence, which returned to control levels by 42 days post-exposure (Table 1). A significantly greater mass dose of FCB (130 fold higher) was required to produce comparable zymosan-stimulated chemiluminescence levels as elicited by UFCB exposure at all post-exposure times (Table 2). However, when dose was normalized to surface area of particles administered, UFCB zymosan-stimulated chemiluminescence levels were only 1.2 to 1.8-fold higher than FCB zymosan-stimulated chemiluminescence levels (Table 2). On a mass dose basis, in regards to NO-dependent chemiluminescence levels, UFCB was 68 times more potent than FCB at 1 day post-exposure and was 130 fold more potent at 7 and 42 days post-exposure. However, when dose was normalized to surface area of particles administered, UFCB NO-dependent chemiluminescence levels were only 1.5-fold greater than FCB at 1 day post-exposure and 3 fold greater at 42 days post-exposure (Table 2). When the surface area zymosan-stimulated or NO-dependent chemiluminescence dose-response curves were analyzed using a linear regression curve with a 95% confidence interval (data similar to figure 2 not shown), there was no significant difference between the UFCB and FCB dose-response curves at any post-exposure time point.

### Comparison of ultrafine carbon black and ultrafine titanium dioxide potency over 42 days

**Figure 3:**
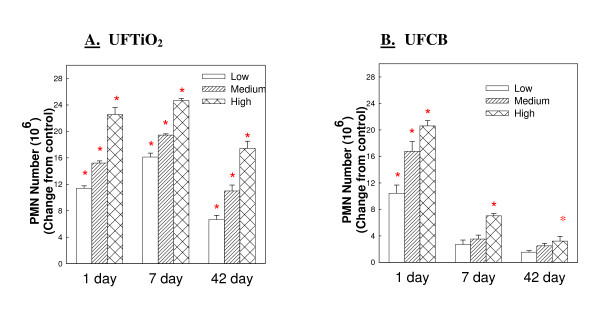
**Comparison of inflammatory potential of low, medium and high doses of UFTiO_2 _(Panel A) and UFCB (Panel B) at the various post-exposure time points**. A comparison of inflammatory potential of low, medium and high doses of UFTiO_2 _(Panel A) and UFCB (Panel B) at the various post-exposure time points. Rats were exposed to various mass doses of UFTiO_2 _(0.26, 0.52 and 1.04 mg/rat) and UFCB (0.047, 0.094, and 0.188 mg/rat) by intratracheal instillation. These mass doses resulted in identical surface area doses (0.0313, 0.0625, and 0.125 cm^2^/cm^2^) of UFTiO_2 _and UFCB. Animals were euthanized at 1 day, 7 days, and 42 days post-exposure and bronchoalveolar lavage was conducted. Inflammation was assessed by measuring PMN levels. Control PMN values were 1.37 × 10^6^, 0.78 ± 0.074 × 10^6^, 0.88 ± 0.095 × 10^6 ^cells/rat for the UFTiO_2 _study and 1.45 ± 0.22 × 10^6^, 1.09 ± 0.14 × 10^6^, and 1.01 ± 0.14 × 10^6 ^cells/rat for the UFCB study at 1, 7 and 42 days, respectively. Values are the increases in PMN number above BALF control and are given as means ± SEM of 8 rats. * indicates that PMN levels for that group were significantly higher than control (p < 0.05) using ANOVA.

Both UFTiO_2 _and UFCB caused a dose dependent increase in lavagable PMN. At 1 day post-exposure the inflammatory activity of UFTiO_2 _(Figure 3 Panel A), indicated by PMN number, is similar to the inflammatory potential of equivalent surface area doses of UFCB (Figure 3 Panel B). However, at 7 days post-exposure, the PMN number elicited by UFCB exposure begins to decrease substantially and continues to decrease over the 42 day post-exposure time period. In contrast, PMN infiltration in response to UFTiO_2 _increased slightly from day 1 to day 7 post-exposure and only slightly decreased at the 42 days post-exposure time period (Figure 3 Panel A).

**Figure 4:**
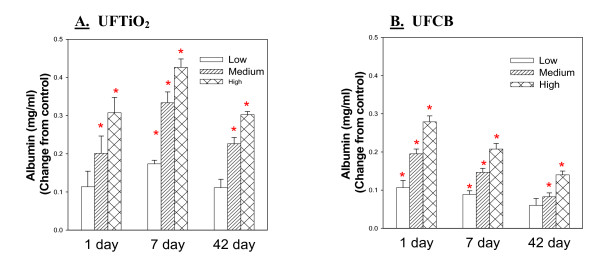
**A comparison of air/blood barrier injury induced by low, medium and high doses of UFTiO_2 _(Panel A) and UFCB (Panel B) at the various post-exposure time points**. Comparison of air/blood barrier injury induced by low, medium and high doses of UFTiO_2 _(Panel A) and UFCB (Panel B) at the various post-exposure time points. Rats were exposed to various mass doses of UFTiO_2 _(0.26, 0.52, and 1.04 mg/rat) and UFCB (0.047, 0.094, and 0.188 mg/rat) by intratracheal instillation. These mass doses resulted in identical surface area doses (0.0313, 0.0625, and 0.125 cm^2^/cm^2^) of UFTiO_2 _and UFCB. Animals were euthanized at 1 day, 7 days, and 42 days post-exposure, and bronchoalveolar lavage was conducted. Air/blood barrier injury was assessed by measuring BALF albumin levels. Control values of albumin were 0.073 ± 0.033, 0.084 ± 0.003, and 0.098 ± 0.007 mg/ml for the UFTiO_2 _study and 0.205 ± 0.11, 0.105 ± 0.010, and 0.184 ± 0.016 mg/ml for the UFCB study at 1, 7 and 42 days, respectively. Values are the increases in albumin levels above the BALF control and are given as means ± SEM of 8 rats. * indicates that albumin levels for that group were significantly higher than control (p < 0.05) using ANOVA.

The same trend is seen when comparing the BALF albumin levels elicited by UFCB and UFTiO_2 _exposure. Albumin levels for equivalent surface area doses of UFCB and UFTiO_2 _are similar at 1 day post-exposure. However, for UFTiO_2 _the albumin levels continue to increase slightly at 7 days and then decrease slightly back to 1 day levels at 42 days post-exposure. For UFCB exposure, albumin levels at 7 days begin to decrease and continue to decrease through the 42 day post-exposure time period (Figure 4).

**Figure 5:**
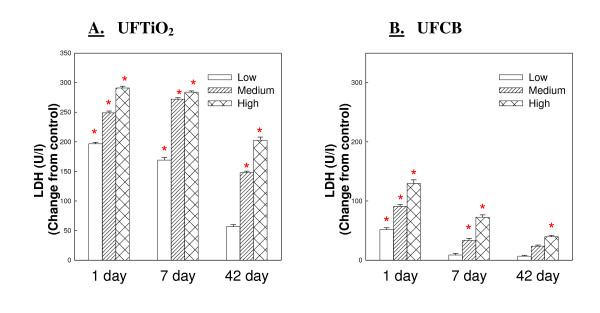
**A comparison of cytotoxicity of low, medium and high doses UFTiO_2 _(Panel A) and UFCB (Panel B) at the various post-exposure time points**. Comparison of cytotoxicity of low, medium and high doses UFTiO_2 _(Panel A) and UFCB (Panel B) at the various post-exposure time points. Rats were exposed to various mass doses of UFTiO_2 _(0.26, 0.52, and 1.04 mg/rat) and UFCB (0.047, 0.094, and 0.188 mg/rat) by intratracheal instillation. These mass doses resulted in identical surface area doses (0.0313, 0.0625, and 0.125 cm^2^/cm^2^) of UFTiO_2 _and UFCB. Animals were euthanized at 1 day, 7 days, and 42 days post-exposure and bronchoalveolar lavage was conducted. Cytotoxicity was assessed by measuring LDH activity in BALF. Control values of LDH activity for the UFTiO_2 _study were 46.375 ± 2.24, 39.5 ± 1.35 and 37.25 ± 2.63 and for the UFCB study were 49.375 ± 1.46, 43.75 ± 0.840 and 45.125 ± 1.69 at 1, 7 and 42 days, respectively. Values are the increases in LDH activity above the BALF control and are given as means ± SEM of 8 rats. * indicates that LDH activity for that group was significantly higher than control groups (p < 0.05) using ANOVA.

LDH activity in BALF elicited by UFCB was lower than LDH activity elicited by UFTiO_2 _at all post-exposure time points. For UFCB, LDH activity was highest at one day post-exposure and decreased throughout the 42 day post-exposure time period. LDH activity for UFTiO_2 _increased at 1 day post-exposure, was sustained at 7 days post-exposure, and only began to decrease slightly at 42 days post-exposure (Figure 5).

## Discussion

The proposed adverse health effects associated with the inhalation of airborne ultrafine or nanoparticles from particulate matter air pollution or from exposure to engineered nanoparticles are topics of ongoing scientific and public concern [16,17]. For analyzing the possible toxicity of nanoparticles, it is necessary to utilize a dose metric that will accurately assess the particles' potential to cause a change in toxicity parameters. Previous toxicity studies have emphasized mass of particles administered as the primary dose metric. However, data from the present study indicate that, for low toxicity low solubility materials, surface area of particles administered appears an appropriate dose metric in studies assessing pulmonary toxicity of nanoparticles.

Data from the present study indicate that when carbon black particles are administered on a mass dose basis, UFCB particles have a significantly greater potential to cause pulmonary inflammation and damage than FCB particles of the same composition. However, when doses of the ultrafine and fine carbon black are normalized to surface area of particles administered, the difference in responses of the two particle sizes becomes non-significant. Our laboratory has reported similar results when comparing pulmonary response to ultrafine and fine titanium dioxide [18]. This, therefore, indicates that, for low toxicity low solubility materials, surface area of particles rather than mass maybe an appropriate dose metric when analyzing nanoparticle toxicity.

A study conducted by Donaldson et al. [10], compared the toxicity of ultrafine and fine particles. UFCB (14 nm) and FCB (260 nm) were instilled into rat lungs based on an equal mass dose. BAL was performed at 6 hours post-exposure and inflammation was assessed by measuring PMN number. Donaldson et al. [10] showed that on a mass basis UFCB caused substantially more inflammation than FCB. In a recent study by Duffin et al. [19], the inflammatory potential of UFCB and UFTiO_2 _was greater than the fine size analogs on an equivalent mass basis. However, the potency of the ultrafine and fine particles was found to be similar when the intratracheally instilled dose to rats was normalized to equivalent particle surface area delivered. Recently, a study by Stoeger et al. [20] utilized six different types of ultrafine carbon particles. The BET-specific surface areas ranged about 35 to 800 m^2^/g, and the mean particle sizes ranged from 10 to 45 nm. This study found that, while the inflammatory potential of these six different particles was dependent on particle type, potency was most strongly related to the BET surface area [20]. These findings support the results of the current study. The results indicate that particle surface area is a critical driver of the cytotoxic and inflammatory potential of nanoparticles. In contrast, Warheit et al. [8,9] reported that intratracheal instillation of fine or ultrafine TiO_2 _resulted in similar levels of acute inflammation and cytotoxicity. They argued that these results were at variance with the "conventional wisdom" that nano-sized particles, due to their larger surface area, are more toxic than fine-sized particles of the same composition. These results conflict with a study from our lab comparing the pulmonary response over a 42 day post-exposure period to intratracheal instillation of ultrafine vs fine TiO_2 _[18]. Results of this study are very similar to the current study with CB, indicating that ultrafine TiO_2 _was more potent that fine TiO_2 _on a mass dose basis, but potencies were similar when exposure was expressed on an equivalent surface area of particles delivered basis. The failure of Warheit and colleagues to observe this relationship is most likely due to severe agglomeration of UFTiO_2 _in their studies, as noted by their dynamic light scattering data indicating that both fine and ultrafine TiO_2 _suspended in PBS exhibited a mean diameter in excess of 2 μm. In contrast, with the current study and that by Sager et al. [18] with TiO_2_, particles were suspended in BALF, which greatly improved dispersion and, thus, effective particle surface area delivered [21]. Indeed, Shvedova et al. [22] reported that improved dispersion of ultrafine carbon black (UFCB) particles in BAL fluid increased the inflammatory and damage potency compared to UFCB suspended in PBS; i.e., intratracheal instillation of a 30 fold greater mass of poorly dispersed UFCB suspended in PBS was required to attain the same level of pulmonary damage (LDH activity) and inflammation (PMN level) as well dispersed UFCB suspended in BAL fluid in a rat model.

As noted above, suspension of UFCB and UFTiO_2 _in BAL fluid greatly improved dispersion of these nanoparticles. Evidence cited above indicates that improved dispersion increases the bioactivity of tested nanoparticles. An issue is whether a poorly dispersed or a well dispersed nanoparticle suspension more closely represents aerosol sizes generated in the workplace. Data from our laboratory indicate that suspension of UFTiO_2 _in BAL fluid results in a mean particle diameter of 204 nm, which is close to the count mode aerodynamic diameter for UFTiO_2 _(138 nm) generated from a dry bulk sample by an acoustical generator [23,24]. Furthermore, structure sizes of multi-walled carbon nanotubes (MWCNT) determined by electron microscopic analysis of MWCNT suspended in a dispersion medium which mimics BAL fluid closely resemble structures generated from a dry bulk sample by an acoustical generator [23,25]. In addition, structure morphology of a MWCNT aerosol produced by this acoustical generator closely resembles that reported in a workplace during production and use of MWCNT [2]. Therefore, it appears that use of well dispersed nanoparticle suspensions for evaluation of bioactivity is relevant for risk assessment of workplace exposures.

In this study, BAL fluid, i.e., diluted alveolar lining fluid was used to effectively disperse UFCB and UFTiO_2_. Porter et al. [23] reported the use of dipalmitoyl-sn-glycero-3-phosphocholine (DPPC) and serum albumin in phosphate-buffered saline at concentrations found in BAL fluid was also an effective dispersant for UFCB, UFTiO_2_, and MWCNT. In contrast, DPPC alone was found to be an ineffective dispersant [21,26]. Recently, Hessemann [27] reported that serum albumin in phosphate-buffered saline was an effective dispersion medium for UFTiO_2_. However, concentrations of albumin required when used alone rather than in combination with DPPC are likely high enough to substantially coat the nanoparticles, which may alter surface activity. In contrast, the combination of DPPC and albumin at the low levels found in the BAL fluid did not alter the bioactivity of crystalline silica in a rat or mouse model [21,23].

The post-exposure time point comparison of equivalent particle surface area doses of UFCB and UFTiO_2 _indicates that at 1 day post-exposure UFCB and UFTiO_2 _produce similar inflammation, cytotoxicity, and air/blood barrier damage. However, as post-exposure time period increases, the inflammation and lung injury caused by UFTiO_2 _was sustained, while that for UFCB tended to resolve. This indicates that UFTiO_2 _causes more persistent pulmonary toxicity than UFCB exposure. Such persistent pulmonary responses to UFTiO_2 _lead NIOSH to recommend an exposure standard for UFTiO_2 _which was significantly lower than that for fine TiO_2 _[4].

## Conclusion

These results support the hypothesis that, for low toxicity low solubility materials, surface area of particles administered, not mass of particles, maybe a more appropriate dose metric to assess to pulmonary inflammation nanoparticles. In addition, the data suggest that on an equivalent surface area of particles delivered basis ultrafine titanium dioxide appears more bioactive than ultrafine carbon black.

## Methods

### Animals for in vivo exposures

The rats used for the in vivo experiments were male Fischer CDF (F344/DuCrl) rats weighing 200–300 g (~10 weeks old at arrival) obtained from Charles Rivers (Raleigh, NC). The animals were housed in an AAALAC-accredited; specific pathogen-free, environmentally controlled facility. The animals were monitored to be free of endogenous viral pathogens, parasites, mycoplasms, Helicobacter and CAR Bacillus. Animals were housed in ventilated cages which were provided HEPA-filtered air, with Alpha-Dri virgin cellulose chips and hardwood Beta-chips used as bedding. The rats were maintained on a ProLaB 3500 diet and tap water, both of which were provided ad libitum.

### Bronchoalveolar fluid collection for particle suspension media

Rats were euthanized with an i.p. injection of sodium pentobarbital (> 100 mg/kg body weight) and exsangainated by cutting the descending aorta. A tracheal cannula was inserted and bronchoalveolar lavage was conducted [28]. A 6 ml aliquot of cold Ca^+2 ^and Mg^+2^-free phosphate-buffered saline (PBS) was used for the lavage wash. The cold PBS was flushed into and out of the lungs two times before the bronchoalveolar lavage (BAL) was collected. The BAL from five rats was combined and centrifuged at 600 × g for 10 minutes using a Sorvall RC 3B Plus centrifuge (Sorvall Thermo Electron Corporation, Asheville NC). The supernatant was decanted into a new tube while the pellet was discarded. This BAL fluid was then used as the vehicle for particle suspensions. The BAL fluid was collected fresh the same day that the particulate suspensions were made.

### Particles

Ultrafine carbon black (UCB – Printex 90, primary particle size = 14 nm), fine carbon black (FCB-Arosperse 15 V, primary partial size = 260 nm) and ultrafine titanium dioxide (UFTiO_2_-Aeroxide TiO_2 _P-25, primary partial size = 21 nm; an 80/20 mixture of anatase/rutile) were obtained as a gift from the Degussa Corporation (Parsippany, NJ).

### Suspension of UFCB, FCB, and UFTiO_2_

UFCB, FCB, and UFTiO_2 _were suspended in rat BAL fluid as described previously [21]. Briefly, each particle sample was sieved using a Retsch AS 200 Sieve (Retsch GmbH, Haan, Germany) through 1.18 mm, 250 μm, and 45 μm mesh screens, and particle samples were weighed to desired amounts. Each respective particle sample was then suspended in the fresh rat BAL fluid to obtain the desired concentration (mg/ml). Once the particles were added to the suspension media, the suspensions were pulse sonicated with 5 individual pulses at a duty cycle setting of 10% and an output setting of 1 with a Branson 450 Sonifier probe sonicator (Branson Ultrasonics Corporation, Danbury, CT). This method has been reported previously to result in well dispersed suspensions as determined by light and electron microscopy [21]. In a previous study, our laboratory evaluated the diameter of UFCB and UFTiO_2 _structures after suspension in BAL fluid using the dynamic light scattering (DLS) technique. Mean diameter of well dispersed UFCB was 131 ± 4 nm, while UFTiO_2 _was 204 ± 18 nm [23]. Microscopically, FCB dispersed in BAL fluid exhibited structures less than 1 μm in diameter on average [21].

### In vivo exposures

To receive their respective dose of particles, each rat was anesthetized with an intraperitoneal (i.p.) injection of methohexital sodium (30–40 mg/kg body weight; Monarch Pharmaceuticals, Bristol, TN). Each animal was then instilled via intratracheal instillation (IT) using a 20-gauage 4-inch ball tipped animal feeding needle. Each animal was instilled with 0.3 ml of their respective dose of UFCB, FCB or UFTiO_2_.

### Bronchoalveolar lavage and cell differentials

At 24-hours, 7 days, and 42 days post-IT, the animals were euthanized with an i.p. injection of sodium pentobarbital (> 100 mg/kg body weight) and exsanguinated by cutting the descending aorta. A tracheal cannula was inserted and bronchoalveolar lavage (BAL) was conducted [28]. A 6 ml aliquot of cold Ca^+2 ^and Mg^+2 ^free PBS was used for the first lavage wash. The cold PBS was flushed into and out of the lungs two times before the BAL fluid was collected. After the first lavage wash was collected, the BAL continued with 8 ml aliquots of cold Ca^+2 ^and Mg^+2^-free PBS until an additional 80 ml of BAL was collected. The BAL from all rats was then centrifuged at 600 × g for 10 minutes using a Sorvall RC 3B Plus centrifuge (Sorvall Thermo Electron Corporation, Asheville NC). After centrifugation, the supernatant from the first lavage wash was decanted into a clean conical vial and stored on ice to be used for cytotoxicity analysis. The remaining lavage wash supernatants were discarded, and the cells remaining were washed with cold Ca^+2 ^and Mg^+2^-free PBS and spun again at 600 × g for 10 minutes. After this, the supernatant was discarded, and the cells were resuspended in 1 ml of HEPES-buffered medium.

Using these BAL cell samples, counting of polymorphonuclear neutrophils (PMN) and alveolar macrophages (AM) was conducted to assess inflammation. The number of AM and PMN was determined according to their unique cell diameters, using an electronic cell counter equipped with a cell sizing attachment (Beckman Coulter Multisizer 3 Counter, Hialeah, FL).

### BAL fluid lactate dehydrogenase activity and albumin concentration

The degree of cytotoxicity induced by the instilled particles was determined by lactate dehydrogenase (LDH) activity in the BAL fluid. LDH activity was measured using Roche COBAS MIRA Plus chemical analyzer (Roche Diagnostic Systems Inc., Branchburg, NJ) as described previously by our laboratory [28]. Albumin concentrations were assessed to examine if instilled particle exposures had compromised the integrity of the alveolar air/blood barrier. The air/blood barrier damage was determined by the concentration of albumin in the BAL fluid. Albumin concentrations were also measured using a Cobas Fara II Analyzer (Roche Diagonostic Systems, Montclair, NJ) as previously described by our laboratory [28].

### Mediator measurements in bronchoalveolar lavage fluid

The presences of inflammatory mediators present in the BAL fluid were analyzed by enzyme-linked immunosorbent assay (ELISA). The levels of mediators present were measured using commercially available ELISA kits (BioSource International Inc., Camarillo, CA). Three mediators were quantified: tumor necrosis factor-α (TNF-α), interleukin (IL)-β, and macrophage-inflammatory protein-2 (MIP-2).

### Zymosan-stimulated and NO-dependent alveolar macrophage chemiluminescence

Reactive oxygen species production was determined by measuring AM chemiluminescence. According to Van Dyke et al. [29], only AM will generate reactive oxygen species in response to unopsonized zymosan in the chemiluminescence assay procedure. The AM chemiluminescence assay was conducted in the same manner as previously described by our laboratory [28]. Briefly, resting AM chemiluminescence was determined by incubating 1.0 × 10^6 ^AM/ml at 37°C for 20 minutes, followed by the addition of 5-amino-2,3-dihydro-1,4, phthalazinedione (luminol) to a final concentration of 0.08 μg/ml. This was then followed by the measurement of chemiluminescence for 15 minutes at 37°C.

Zymosan-stimulated chemiluminescence (CL) was determined by adding unopsonized zymosan (2 mg/ml) to the AM samples immediately prior to measurement of chemiluminescence. Zymosan-stimulated CL was calculated as (CL with zymosan – resting CL). NO-dependent chemiluminescence was determined by adding the unopsonized zymosan as well as N-nitro-L-arginine methyl ester HCL (L-NAME) to the AM samples immediately prior to measurement of chemiluminescence. NO-dependent CL was calculated as (zymosan-stimulated CL without L-NAME – zymosan-stimulated CL with L-NAME). Zymosan-stimulated and NO-dependent chemiluminescence were both measured using a Berthold automated luminometer (Berthold Autolumat LB 953, EG&G, Gaithersburg, MD) at 390–620 nm for 15 minutes.

### Statistics

Statistical differences between control groups and treatment groups for the in vivo experiments examining the toxicity of carbon black and titanium dioxide were determined using an analysis of variance (ANOVA) with significance set at p ≤ 0.05. Individual means were compared using the Student-Newman-Keuls Method multiple comparison procedure with an overall significance level of p ≤ 0.05. In addition, linear regression curve analysis with a 95% confidence interval was conducted on the surface area data of each pulmonary parameter measured.

## Abbreviations

AAALAC: Association for Assessment and Accreditation of Laboratory Animal Care; AM: Alveolar macrophage; ANOVA: Analysis of variance; BAL: Bronchoalveolar lavage; BALF: Bronchoalveolar lavage fluid; BET: Brunauer-Emmett-Teller; Ca^+2^: Calcium; CAR: Cilia-associated-respiratory; CB: Carbon black; cm: centimeter; FCB: Fine carbon black; IL: Interleukin; i.p.: intraperitoneal; IT: Intratreacheal instillation; LDH: Lactate dehydrogenase; Mg^+2^: Magnesium; μm: Micrometer; mg: Milligram; MIP: Macrophage inflammatory protein; ml: Milliliter; NF: Nuclear factor; nm: Nanometer; OEL: Occupational exposure level; PBS: Phosphate buffered saline; PM: Particulate matter; PMN: Polymorphonuclear leukocyte; SE: Standard error; TNF: Tumor necrosis factor; UF: Ultrafine; UFCB: Ultrafine carbon black; UFTiO_2_: Ultrafine titanium dioxide.

## Competing interests

The authors declare that they have no competing interests.

## Authors' contributions

TMS carried out all of the in vivo experiments involved in this study including the intratracheal instillations and animal sacrifices. TMS drafted the manuscript and performed the statistical analysis. Both TMS and VC conceived of the study and participated in its design. VC participated in the study coordination, data analysis and interpretation, and helped draft the manuscript. All authors read and approved the final manuscript.
